# Conservation hints for *Pinna nobilis* from a century-old genetic time capsule

**DOI:** 10.1038/s41598-025-21574-6

**Published:** 2025-10-28

**Authors:** Ilenia Azzena, Chiara Locci, Noemi Pascale, Ilaria Deplano, Riccardo Senigaglia, Edoardo Batistini, Daniela Caracciolo, Mariachiara Chiantore, Saul Ciriaco, Maria Paola Ferranti, Daniele Grech, Arianna Liconti, Monica Montefalcone, Alice Oprandi, Valentina Pitacco, Marco Segarich, Rym Zakhama-Sraieb, Ahmed Ben Hmida, Salma Zribi, Fabio Scarpa, Marco Casu, Daria Sanna

**Affiliations:** 1 Department of Veterinary Medicine, University of Sassari, Via Vienna 2, 07100 Sassari, Italy; 2 Department of Biomedical Sciences, University of Sassari, Viale San Pietro 43B, 07100 Sassari, Italy; 3 Department of Chemical, Physical, Mathematical, and Natural Sciences, University of Sassari, Via Vienna 2, 07100 Sassari, Italy; 4 Shoreline Soc. Coop., AREA Science Park, Località Padriciano 99, 34149 Trieste, Italy; 5 ARPAL, Regional Agency for the Environmental Protection Liguria, Genova, Italy; 6 DiSTAV (Department of Earth, Environment and Life Sciences), University of Genoa, Corso Europa, 26, 16132 Genova, Italy; 7 National Biodiversity Future Center (NBFC), Palermo, Italy; 8 WWF Fondazione, Area Marina Protetta Di Miramare, Via Beirut 2/4, 34151 Trieste, Italy; 9 IMC - International Marine Centre, Loc. Sa Mardini, 09170 Torregrande, Oristano, Italy; 10 OutBe, Via Nino Bixio 19, 16043 Chiavari, Genova, Italy; 11 Marine Biology Station Piran, National Institute of Biology, Fornače 41, 6330 Piran, Slovenia; 12 Institut Supérieur de Biotechnologie de Sidi Thabet, University of Manouba, Sidi Thabet, Tunisia; 13 Coastal Protection and Development Agency (APAL), 5000 Monastir, Tunisia; 14 Faculty of Sciences of Tunis, Laboratory of Diversity, Management and Conservation of Biological System, University of Tunis El Manar, LR18ES06, Tunis, Tunisia

**Keywords:** Evolution, Genetics, Zoology

## Abstract

The noble pen shell, *Pinna nobilis,* is an iconic marine bivalve endemic to the Mediterranean Sea, playing a key role as an ecosystem engineer. Over the past century, it has faced severe threats from overharvesting, pollution, and catastrophic mass mortality events. This study analysed 119 mitochondrial COI gene sequences from historical (1700s, 1920s, 1970s, 1990s) and modern (2000s) samples, including survivors of recent mass mortality crises. We standardised a protocol to extract DNA from ancient byssus samples over a century old and dated the emergence of the mitochondrial lineages of *Pinna nobilis*, uncovering its evolutionary history in unprecedented detail. Our findings suggest two main temporal origins for the species’ genetic variation: (i) a group of modern lineages directly descended from *Pinna nobilis* early ancestors originating 2.5 mya, and (ii) a large group derived from the first Pleistocene radiation of the species, approximately 1.5 mya. Importantly, our research depicts the evolutionary response of *Pinna nobilis* to three major challenges in the last century: human overexploitation, pollution, and environmental changes. Our results highlight the species’ remarkable resilience, likely mediated by Pleistocene genetic traits, whose persistence over time mainly depends on the maintaining of a high effective population size to ensure successful recruitment.

**Supplementary Information:**

The online version contains supplementary material available at 10.1038/s41598-025-21574-6.

## Introduction

*Pinna nobilis* (Linnaeus, 1758), known as the noble pen shell, is a key species endemic to the Mediterranean Sea. It belongs to the family Pinnidae (Mollusca: Bivalvia)^1^ and is commonly found in coastal waters at depths between 0.5 and 60 m, favouring soft-bottom habitats, especially those with mixed seagrass meadows. It is primarily associated with *Posidonia oceanica* beds, where its filter-feeding activity contributes significantly to ecosystem functioning^1^.

*Pinna nobilis* supports marine ecosystems by providing surfaces for organisms to attach to, filtering up to 2500 Liters of water daily, and sustaining symbiotic crustaceans and local food webs^1–3^*.* Historically, *Pinna nobilis* was renowned for its byssus, used to produce the sea silk (a luxurious textile), as well as its muscle (a delicacy) and decorative shell. Overharvesting for these purposes led to a decline in *Pinna nobilis* populations during the twentieth century^1,4,5^.

To protect the species, a complete ban on harvesting was introduced in 1992 across European countries (Annex IV of European Council Directive 92/43/EEC). This strict protection regime led to a gradual increase in population size in the Mediterranean. As a result of this recovery, researchers were able to investigate the genetic variability of *Pinna nobilis* throughout the basin^6–14^. Initial studies on *Pinna nobilis* focused on mitochondrial markers^6,7,11^ revealing low genetic differentiation among populations in the Eastern Mediterranean, suggesting a lack of strong genetic structuring in that area^6^. Subsequent broader assessment across the Mediterranean^11^ identified patterns of genetic variability that appeared to follow three main marine ecoregions: (1) the western Mediterranean and Ionian Sea, (2) the Adriatic Sea, and (3) the Aegean Sea along with the Tunisian coasts. However, a more recent study by Sanna et al.^14^, which included a significantly larger number of samples from the previously underrepresented Adriatic Sea, revealed genetic homogeneity between Adriatic and Western Mediterranean populations, indicating a higher degree of interregional connectivity than previously thought.

Later genetic research incorporated microsatellite markers^8–10,12,13^, sometimes in combination with mitochondrial data^8^, and confirmed both a general high genetic variability for the species and low inter-population differentiation. These findings support the existence of a single genetic lineage that likely experienced a recent population expansion^10^. This hypothesis is further supported by the most recent work by Sanna et al.^14^, which suggests that *Pinna nobilis* diverged from an Atlantic ancestor approximately 2.5 million years ago, likely colonizing the Western Mediterranean following the Zanclean flood. The species then expanded into the Adriatic and Eastern Mediterranean, shaping its current genetic distribution and structure.

Despite the recovery experienced in the early 2000’s, *Pinna nobilis* populations faced a new threat in 2016 in the form of Mass Mortality Events (MMEs), which severely reduced their numbers. These events began along the Spanish coasts and spread across the Mediterranean^15^. They were initially attributed to the parasite *Haplosporidium pinnae*^16^ however, further research revealed that the die-offs were likely driven by a complex interplay of pathogens, including bacteria and viruses, compounded by environmental stressors^17–21^. As populations declined, the surviving *Pinna nobilis* individuals were confined to isolated refuges in coastal lagoons and estuaries across the coastlines of Spain, France, Italy, Greece, and Turkey^22–31^. However, the reasons why certain populations have managed to survive, while others have not, remain unclear, warranting further investigation.

In response to this crisis, large-scale conservation projects were launched, with the European LIFE PINNA project standing out. This effort aims to protect the remaining populations and restore affected areas through measures such as breeding *Pinna nobilis* in captivity and transplanting individuals to safer areas. “Citizen Science” initiatives have also played a key role, with divers and local communities helping locate surviving populations, including a resilient group found near Sant’Antioco Island in Sardinia, Italy (Western Mediterranean).

To gain deeper insights into the species evolutionary history, we analysed ancient byssus samples, obtained through the collaboration of two Sardinian weavers, Assuntina Pes and Giuseppina Pes, who continue to work with the byssus threads from the collection of Italo Diana, one of the most important Sardinian byssus weavers—a legacy of the atelier he founded in the 1920s in Sant’Antioco—and of the so-called “sea silk master” weavers Chiara Vigo. These samples, up to 300 years old, provided a unique opportunity to study how the genetic make-up of *Pinna nobilis* has evolved over time. By comparing ancient samples with those from modern (before MMEs^6,7,10,11,14,24,32,33^) and surviving populations (after MMEs^24,33^), valuable insights into genetic changes and adaptations were gained.

In this context, the focus of this study is twofold: first, it aims to understand the population dynamics of *Pinna nobilis* during the early Pleistocene; second, it examines the impact of human and environmental stressors in the species’ recent history. To enable a comparison among ancient, modern, and surviving populations, these authors relied on the Cytochrome c Oxidase Subunit I (COI) gene as the main tool for this research, as it is the only molecular marker that allows the broadest comparisons with sequences^6,7,10,11,14,24,32,33^ from different areas and time periods.

Through a combination of genetic knowledge and dedicated conservation efforts, there is hope that this iconic species will continue to thrive in the Mediterranean for generations to come.

## Results

### Phylogenetics and phylogeographic analyses

The whole dataset, consisting of 667 sequences, was divided into three main cohorts: ancient populations (1920s, 1970s, 1990s, and one individual from 300 years ago), modern populations (early 2000s, pre-MMEs), and survivor populations (post-MMEs). The 119 newly generated sequences were obtained from three areas: Sant’Antioco Island (Sardinia, Italy), which included ancient, modern and survivor populations; Tunisia and North Adriatic Sea, which included survivor populations.

Genetic variation analysis across the whole Mediterranean dataset revealed 59 polymorphic sites, resulting in 100 haplotypes. Among the three temporal cohorts, the ancient one, exhibited the lowest rates of genetic variation in terms of haplotype (h) and nucleotide diversity (π) (see Table 1). However, it should be taken into consideration that such a reduced diversity in the ancient group may be explained by the fact that modern and surviving cohorts are represented by a larger number of individuals from both eastern and western Mediterranean populations, while the ancient cohort is exclusively represented by samples from one western population.

**Table 1:** Sample sizes and genetic diversity estimates for the mitochondrial COI gene fragment analysed in *Pinna nobilis* individuals from various Mediterranean regions across distinct time periods: N: sample size; S: number of polymorphic sites; H: number of haplotypes; h: haplotype diversity; π: nucleotide diversity. *Ancient dataset from Sant’Antioco also included 1 sample of 300 years ago, which could not be incorporated into the other groups or treated separately as it corresponds to only one individual.

Samples	N	S	H	h	π
Ancients—Sant’Antioco Island (Western Mediterranean)					
1920’	17	2	3	0.412	0.00131
1970’	20	6	6	0.721	0.00366
1990’	6	2	3	0.733	0.00276
Total ancients	44*	7	7	0.613	0.00263
Moderns					
Total moderns	552	55	92	0.892	0.00675
**Survivors**					
Total survivors	71	13	16	0.837	0.00525

In contrast, between the early 2000s (moderns) and 2024 (survivors) (see Table S1 for details), the species has maintained high genetic variation, with a slight decrease in the survivor populations (see Table 1). However, the survivors showed reduced polymorphic sites (S), and haplotypes (H) compared to modern populations, potentially influenced by the larger effective population size of modern individuals.

The ancient cohort from Sant’Antioco Island was categorized into three groups based on the sampling period: 1920s, 1970s, and 1990s. The individuals sampled in 1920s showed the lowest level of genetic variability (see Table 1). In contrast, individuals from the 1970s and 1990s exhibited higher levels of genetic variability, despite fewer available sequences from the 1990s. Notably, individuals from the 1970s displayed the highest genetic variability within the ancient cohort, with six haplotypes identified out of a total of 7 (see Table 1).

**Fig. 1:**
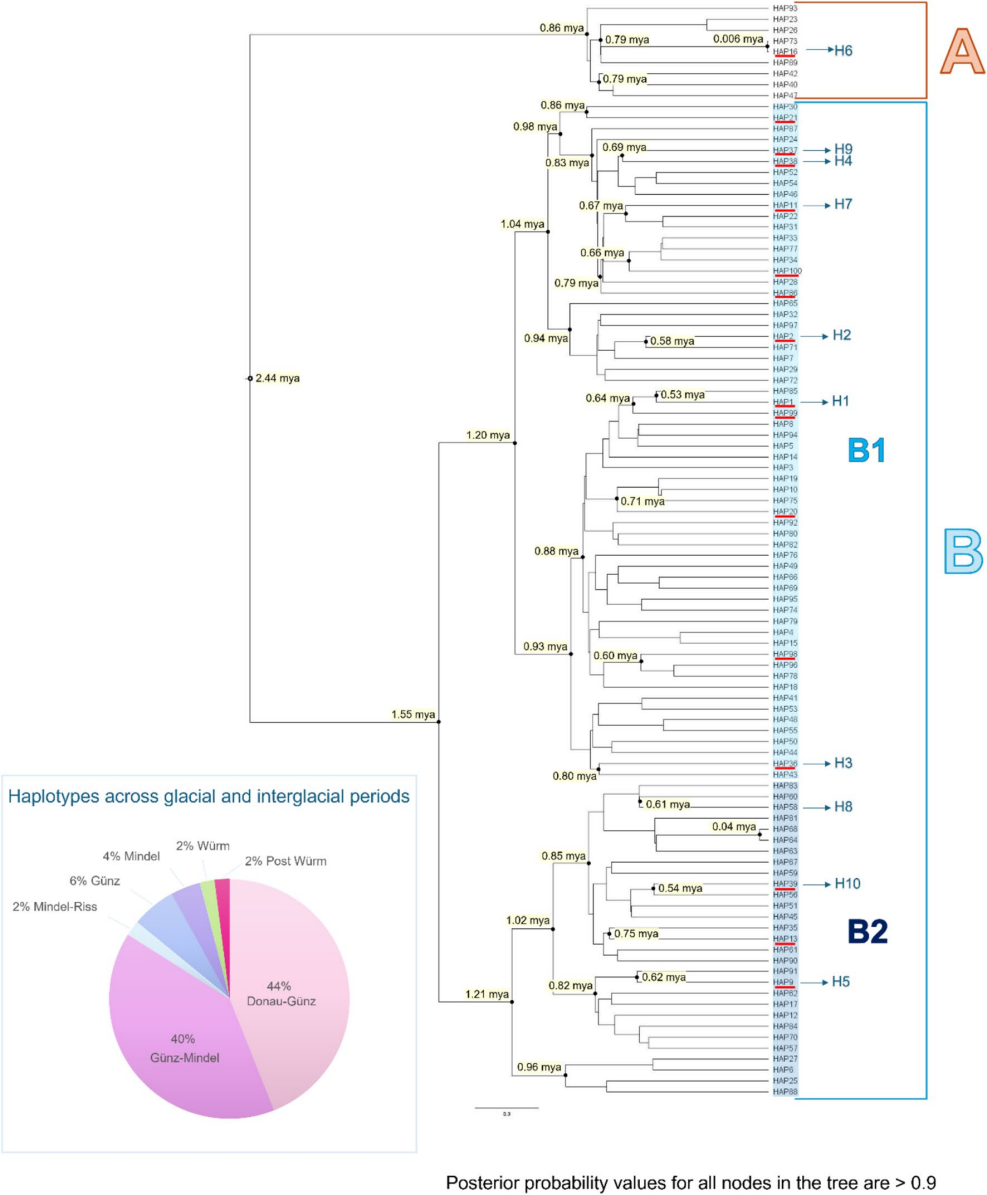
Time-calibrated tree based on the mitochondrial COI gene fragment. All nodes are highly supported with value exceeding 0.9. Node values indicate divergence times, expressed in millions of years ago (mya). The 100 haplotypes are labelled with the code “HAP”. Haplotypes underlined in red represent those still present in the surviving Mediterranean populations. The 10 most common haplotypes are indicated with the code “H”. The inbox in the bottom left shows the percentage of haplotypes that evolved during different time periods.

A subset of 100 sequences, corresponding to all *Pinna nobilis* COI haplotypes identified in this study and shared among ancient, modern, and survivor cohorts, was used to perform a time-calibrated tree analysis (see Fig. 1 and Figure S1 in supplementary materials for details on molecular dating).

The time calibrated tree (see Fig. 1 and Figure S1), generated with BEAST, indicates that the ancestor of *Pinna nobilis* originated in the Mediterranean ~ 2.5 mya.

The common ancestor gave rise to two clades: Clade A and Clade B. Clade A, which diverged ~ 860 kya, comprises 9 of the 100 haplotypes (9%) directly descending from the early ancestor of *Pinna nobilis*. This clade (A) was once widespread across the whole Mediterranean (Western Mediterranean, Adriatic Sea, and Eastern Mediterranean), but most haplotypes seem to have gone extinct after MMEs, or at least they are extremely rare and not detected in the sampled area of this study, except H6 (Fig. 1). H6, the sixth most common haplotype, persists in survivor populations from the Western Mediterranean (Mar Menor, Spain) and North Adriatic Sea (Trieste and Miramare, Italy). Notably, 55.5% of Clade A haplotypes diverged during the Donau-Günz interglacial (949–681 kya; see the red-underlined haplotype in Clade A, Fig. 1). Additionally, H6, along with the modern haplotype from the Western Mediterranean (Isola Piana, Sardinia, Italy) HAP73, are among the most recently originated haplotypes. HAP73, which now could be extinct or extremely rare and not detected in this area of the Mediterranean, and H6, diverged during the post-Würm period ~ 6 kya (see Clade A in Fig. 1).

Clade B includes 91% of all haplotypes and exhibits two main subclades, B1 and B2 (see Fig. 1), that share a common ancestor which originated 1.55 mya. Subclade B1, which differentiated 1.20 mya, comprises 62% of the whole lineages. Within this subclade, two main, almost contemporary subgroups are included. Among them, the subgroup that differentiated 0.93 mya includes the greatest number of haplotypes, but proportionally, it also groups the highest number lineages that could be now extinct or extremely rare.

Subclade B2, which originated 1.21 mya, accounts for 29% of haplotypes. Subclade B1 includes 6 of the 10 most common haplotypes (H1, H2, H3, H4, H7, and H9 in Fig. 1). Among these, haplotypes H3, H4, and H9 diverged during the Donau-Günz interglacial (~ 800 kya for H3, ~ 690 kya for H4 and H9), preceding the first major Quaternary glaciations (Günz, Mindel, Riss, and Würm). Haplotype H7 diverged during the Günz glacial period (~ 670 kya), while the most widespread H1 and H2 arose during the Günz-Mindel interglacial (~ 530 kya and 580 kya respectively). Haplotype H1 was also retrieved in a 300-year-old sample from Sant’Antioco Island (Western Mediterranean), suggesting that it was among the most common haplotypes historically. Moreover, within subclade B1, 12 haplotypes persist in surviving Mediterranean populations (see red-underlined haplotypes in subclade B1, Fig. 1), representing 75% of total surviving haplotypes. Of these 12 haplotypes, 6 are among the most common (H1-H4, H7 and H9 in subclade B1, Fig. 1), with the divergence times previously reported. In particular, haplotypes H2 and H7 are mainly found in the Western Mediterranean and the Adriatic Sea, while H3, H4, and H9 are prevalent in the Eastern Mediterranean and the Ionian Sea. Notably, haplotype H1 is primarily distributed in the Western Mediterranean and Adriatic Sea, but it has also been detected in two survivor individuals from the Tunisian Kerkennah Islands in the Eastern Mediterranean.

Whereas the remaining 6 haplotypes diverged during the following periods: 3 during the Donau-Günz (~ 949–681 kya), 1 during the Günz-Mindel (~ 619–456 kya), and 2 during the Günz (~ 680–620 kya).

Subclade B2 includes the three remaining prevalent haplotypes (H5, H8, and H10, Fig. 1), originating during the Günz-Mindel interglacial period and broadly distributed across the Mediterranean Sea. Among these, haplotypes H5 and H10 persist in surviving populations. Specifically, H5 is found in the Western Mediterranean (Sant’Antioco Island) and the Northern Adriatic Sea, while H10 is distributed in the Ionian Sea (Amvravikikos Gulf, Greece). In contrast, haplotype H8 appears extinct or extremely rare and not detected.

Similar to Clade A, Clade B includes two recently diverged haplotypes, HAP68 and HAP64 (Fig. 1), which diverged during the Würm glacial period (~ 41 kya). These haplotypes, found in modern populations from Sicily and Elba Islands (Western Mediterranean), now appear extinct or extremely rare and not detected.

In summary, most haplotypes identified in *Pinna nobilis* populations across ancient, modern, and surviving individuals diverged during the Donau-Günz (44%) and Günz-Mindel (40%) interglacial periods (see inbox in Fig. 1).

**Fig. 2:**
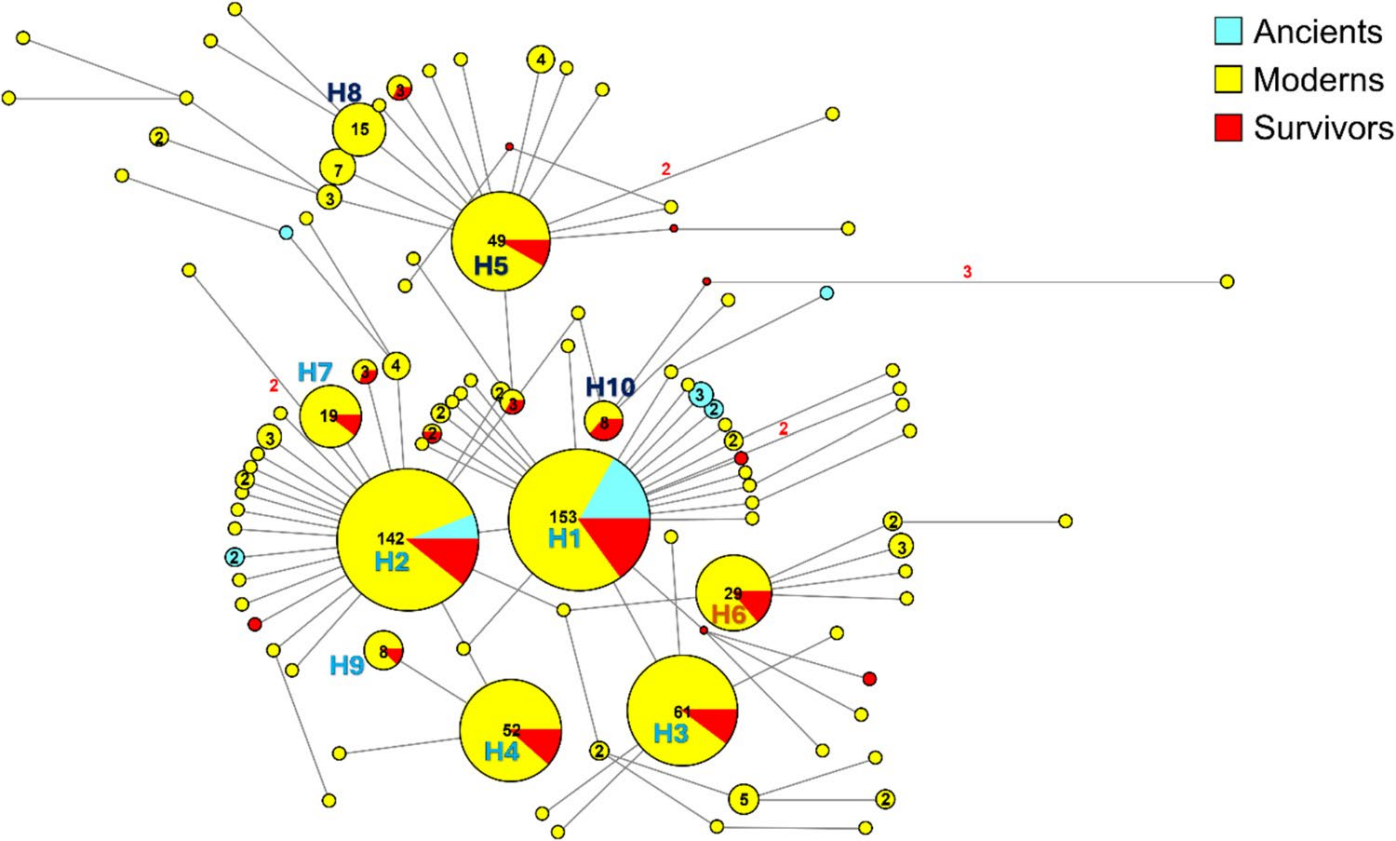
Median-joining network analysis performed on the mitochondrial COI gene fragment for *Pinna nobilis* populations over time, with the resulting haplotypes color-coded based on their sampling period. The small red spots on the nodes show median vectors representing the hypothetical sequences that were calculated using the maximum parsimony method. The number of mutations between haplotypes that are greater than n = 1 are reported on the network branches. Additionally, the number of individuals showing the same haplotype that is greater than n = 1 are reported inside the spot. Haplotype codes (H1–10) are reported on the network with the colours used in the phylogenetic tree (Fig. 1) to indicate Clade A and Subclades B1 and B2.

The network analysis (see Fig. 2) described the phylogeographic patterns of the 100 haplotypes identified within the whole dataset.

Ten of these haplotypes are the most prevalent (H1–H10, Fig. 2), forming at least three main star-like patterns. These ten haplotypes are defined by a total of eight mutations. Of these, seven mutations are silent, resulting in no change to the amino acid sequence. The remaining mutation, located at position 62 of the analysed COI fragment and found exclusively in haplotype H8, is a missense mutation. This substitution leads to a change in the amino acid sequence, replacing glycine with glutamic acid.

The two most common haplotypes, H1 and H2, are shared across all sampled periods—ancient, modern, and survivor—comprising 22.9% and 21.3% of the whole dataset, respectively. In particular, they were present in 79.5% of the ancient cohort. The remaining 20.5% of ancient samples show haplotypes exclusive to this cohort, which probably went extinct in modern and survivor populations. Overall, 71.4% of ancient haplotypes appear to have no remaining descendants in surviving populations.

The two most common haplotypes among the Eastern and Ionian samples (H3 and H4 in Fig. 2) are directly derived from the two most common haplotypes in the dataset (H1 and H2 in Fig. 2).

Haplotypes H3 and H4 differ from H1 and H2 due to two main single-nucleotide polymorphisms (SNPs) within the last 25 nucleotides of the analysed COI fragment. Specifically, one polymorphism is prevalent in nearly all eastern individuals, resulting in a silent mutation at the third base of a codon encoding glycine, involving a transition between the purine bases A and G. This mutation does not cause any amino acid changes in the protein.

**Fig. 3:**
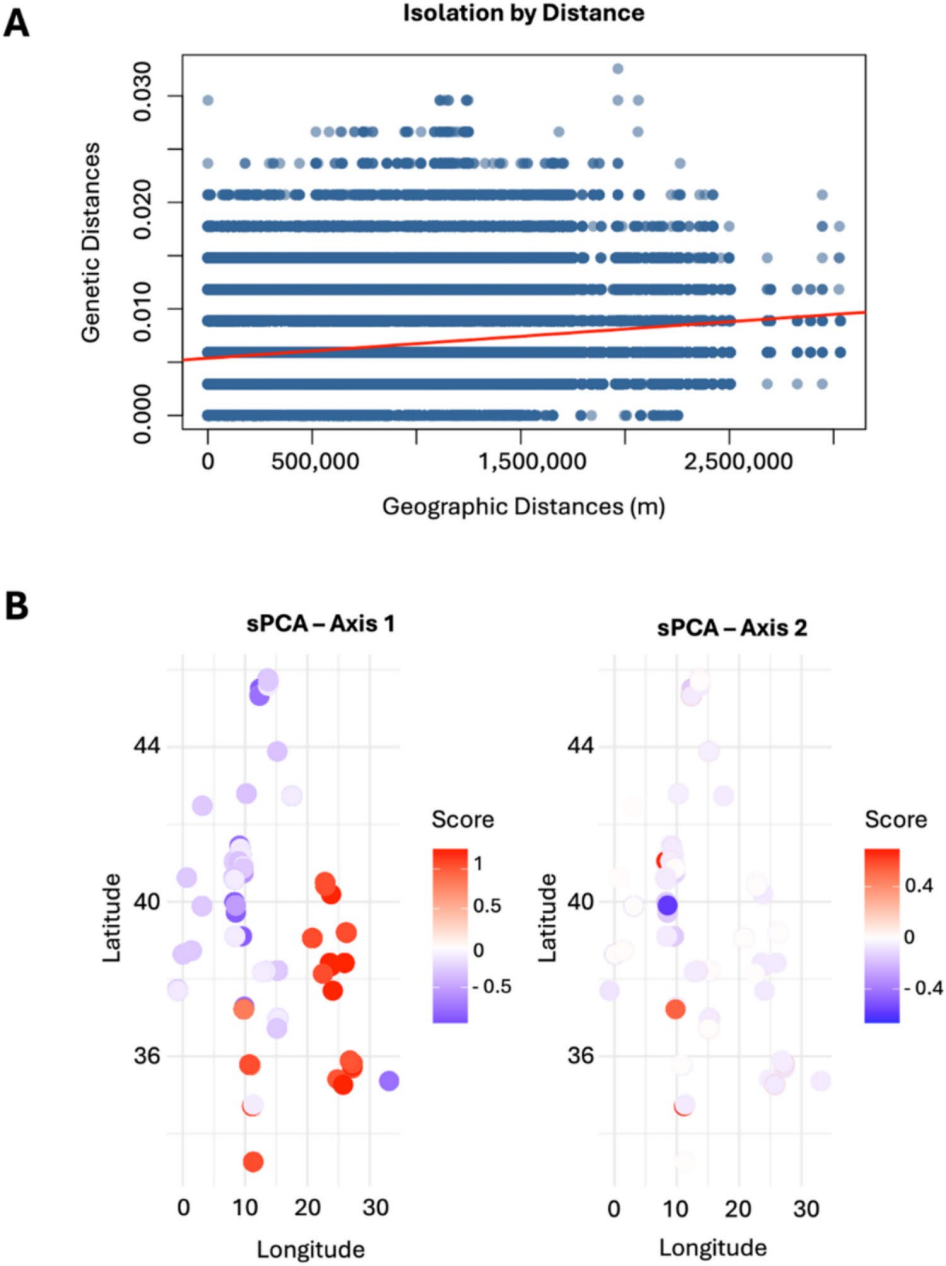
Analysis of spatial genetic structure. (**A**) Plot of the results obtained for the isolation by distance that show the relationship between genetic distance (Y-axis) and geographic distance (X-axis, in meters): the linear regression (red line) indicates a weak, although significant positive correlation. (**B**) Spatial Principal Component Analysis (sPCA). Scores represent combinations of allele frequencies that maximize both genetic variance and spatial autocorrelation.

The Mantel test, used to test the hypothesis of the isolation by distance (IBD) among Mediterranean sites, yielded a correlation coefficient of r = 0.15, with a very high level of significance (p = 9.999 × 10⁻^5^, based on 10,000 permutations). The absolute value of the correlation coefficient (r = 0.15) indicates that the strength of the association is relatively weak (Fig. 3a). This observed value was significantly higher than the quantiles of the null distribution, generated through random permutations of the matrices (i.e. 90% = 0.0289; 95% = 0.0376; 97.5% = 0.0448; 99% = 0.0540). Such a result confirms that the observed correlation between genetic distance and geographic distance is not compatible with a random model that would have supported the presence of IBD among the *Pinna nobilis* sampling sites in the present study: indeed, a non-random spatial structure in the distribution of genetic variability was found for the dataset which was analysed.

Finding obtained by the Mantel test highlight the existence of a weak IBD among Mediterranean sites for *Pinna nobilis* and suggest that factors other than the geographic distance, (e.g., historical events, geographical barriers, demographic or selective effects) account for the observed genetic structure in accordance with a complex biological system subjected to multiple influences.

In this context, Fig. 3b shows how the first two principal axes (85.71% of the total variance explained), obtained for the spatial PCA (sPCA), explain the limited portion of the IBD retrieved by Mantel test (Fig. 3a). Axis 1 (63.5%) reveals a spatial structure with an east–west gradient, suggesting major spatial genetic differentiation. Indeed, this axis highlights a spatial structuring, with a concentration of positive values (in red) in the eastern part of the Mediterranean basin, suggesting significant geographical differentiation among sampling sites, likely due to ecological barriers or environmental factors. In contrast, axis 2 (22.3%) captures a weaker, less structured component of variability, potentially reflecting local processes or stochastic patterns not related to geographic distances. This axis shows weaker and more diffuse variation, indicating potential local or secondary differences not associated with a clear geographical gradient.

## Discussion

This study provides a comprehensive overview of the evolutionary history of the critically endangered Mediterranean bivalve *Pinna nobilis*. By comparing genetic data from ancient, modern, and surviving populations, we examined how genetic diversity, and evolutionary trends have shifted over the past century. Our findings shed new light on how *Pinna nobilis* has managed to survive major environmental changes, human overexploitation, and the most recent MMEs that have dramatically decreased its populations.

In this context, we found that the mitochondrial genetic structure of the species has remained stable, with key lineages (haplotypes) persisting across ancient, modern, and surviving populations, likely because they provide survival advantages.

Furthermore, the analyses conducted in the present study did not detect signals of isolation by distance, corroborating the results obtained in previous studies^10,11,32^ and suggesting effective larval dispersal for *Pinna nobilis*. However, rather than being correlated with geographic distances, the observed genetic structure appears to reflect the evolutionary dynamics resulting from the early Pleistocene radiation of the species, shaped by environmental conditions and the ecological characteristics of fan mussels.

Previous studies suggested that *Pinna nobilis* originated in the Western Mediterranean around 2.5 mya^14^ and began a rapid spread across the basin about 1.5 mya. However, it should be considered that this finding is based on a fragment of the mitochondrial COI gene, and therefore, the evolutionary trend could be modified or implemented by analysing a larger fragment. Nonetheless, the present study corroborates the previous finding^14^ that the Mediterranean expansion process, which preceded the Pleistocene radiation, took about one million years, allowing *Pinna nobilis* ancestor to achieve a stable and well-adapted genetic make-up that persists to the present day.

Examining the temporal origin of the mitochondrial haplotypes, we identified two major genetic groups within *Pinna nobilis*: Clade A and Clade B. These groups trace back to different points in the species’ evolutionary history. Clade A is representative of the species’ early ancestral genetic pool, while Clade B appears to be derived from the first Pleistocene radiation event, reflecting changes that helped the species adapt to new Mediterranean environments, after its origin 2.5 mya. Interestingly, nearly all mitochondrial lineages which characterised the species in the last century diverged between 900 and 500 kya, suggesting that the genetic diversity observed in the 1900s originated in ancient times. The persistence of certain haplotypes over long periods may be linked to their role in enhancing the species’ resilience.

The more common mitochondrial haplotypes found in this study may represent ancient lineages associated with mitochondrial or nuclear genes that likely conferred adaptive advantages during the Pleistocene radiation, such as greater resilience to fluctuations in temperature, salinity, and food availability. These mitochondrial haplotypes may harbour advantageous genetic variants positively selected during glacial and interglacial cycles, enabling *Pinna nobilis* to endure severe bottlenecks caused by pollution, pathogens, environmental changes, and human overexploitation in the twentieth and twenty-first centuries.

For these reasons, the long-term stability of *Pinna nobilis* haplotypes, as highlighted in this study, is a key factor in the species’ resilience and survival. Remarkably, *Pinna nobilis* has exhibited minimal genetic changes over time, indicating that its haplotypes are highly adapted to prevailing environmental conditions, resulting in a state of genetic equilibrium. This stability has likely played a crucial role in the species’ persistence, enabling it to adapt effectively to environmental changes and withstand human-mediated evolutionary pressures.

However, this genetic stability has not made the species invulnerable to different stressors. Over the last century, *Pinna nobilis* populations have faced multiple threats that significantly impacted their genetic variability and survival. One of the earliest pressures was human overexploitation, driven by the demand for sea silk (byssus) production and the harvesting of the shells for ornaments and collectibles^35^. Moreover, its abductor muscle was also consumed as a delicacy^4^. This overexploitation caused a reduction in genetic variability, as random harvesting indiscriminately removed individuals regardless of their haplotype, thereby limiting the haplotype richness of the genetic pool. Later, pollution produced by anthropogenic activities, such as mining and intensive agriculture^36^ introduced additional stressors that likely affected population health and fitness (reproductive success) across the whole Mediterranean basin. This could have affected the size of populations where only individuals with resilient haplotypes survived. Moreover, from 2016 onwards, MMEs linked to a multifactorial disease had a devastating impact on *Pinna nobilis* populations across the Mediterranean, experiencing a sharp decline in population size. This led to the persistence of very few individuals with the most common mitochondrial haplotypes which likely are involved in resilience and capability to survive.

Notably, genetic divergence has remained high, enabling natural selection to act on a broad pool of allelic variants and favour those most beneficial for long-term survival. In this context, while natural bottlenecks (such as those produced by MMEs) reduce population size, they may facilitate the selection and fixation of beneficial traits in remaining populations. In contrast, human-driven bottlenecks (such as overexploitation for sea silk) reduce genetic variability randomly, increasing the risk of losing advantageous genetic traits (and preserving maladaptive traits just by chance) and thus posing a greater threat to long-term adaptability. Furthermore, the beneficial variants that support the species’ persistence are likely not strongly linked to maladaptive ones. This potentially helps preserve their positive effects, allowing the overall advantage to outweigh any potential disadvantages.

Importantly, the study offers a surprising and hopeful discovery: present-day surviving populations exhibit higher genetic variability than those recorded in the early 1900s. This increase in genetic variability may be attributed to the persistence of haplotypes that trace back to ancient lineages. It also evidences that even small “founder populations” (those remained after significant population declines) can retain the genetic potential required for recovery. This finding reinforces the importance of protecting remnant populations (even isolated individuals), as they may harbour the genetic diversity essential for the species’ future survival.

The genetic legacy of *Pinna nobilis* reflects its stability, resilience, and ability to adapt. Despite facing major environmental changes and human impact over thousands of years, its genetic structure has stayed largely unchanged.

Moreover, the dispersal potential of *Pinna nobilis* larvae, aided by surface currents and hydrodynamic factors, promotes connectivity and reinforces genetic flow among populations. In particular, temperature appears to be an important abiotic factor shaping genetic differentiation between eastern and western populations^37^. Variations in temperature can regulate spawning periods and affect larval development and settlement. Warmer waters, typical of the eastern Mediterranean, may lead to earlier spawning and shortened larval durations, promoting settlement closer to parental habitats. In contrast, cooler waters in the western Mediterranean could prolong the planktonic larval phase, allowing for wider dispersal and greater connectivity even among distant areas.

These temperature-driven dynamics, along with ocean currents and other hydrodynamic forces, may therefore help to explain the general genetic homogeneity of *Pinna nobilis* populations in Western Mediterranean and the slight level of isolation by distance highlighted by the Mantel test in the Eastern Mediterranean.

For this reason, conservation efforts should focus on protecting populations by avoiding the harvesting of individuals across the Mediterranean to preserve this diversity.

The persistence of key mitochondrial haplotypes overtime and the retention of genetic diversity in surviving populations offer hope for the recovery of this iconic Mediterranean species.

## Materials and methods

### Sample collection

**Fig. 4:**
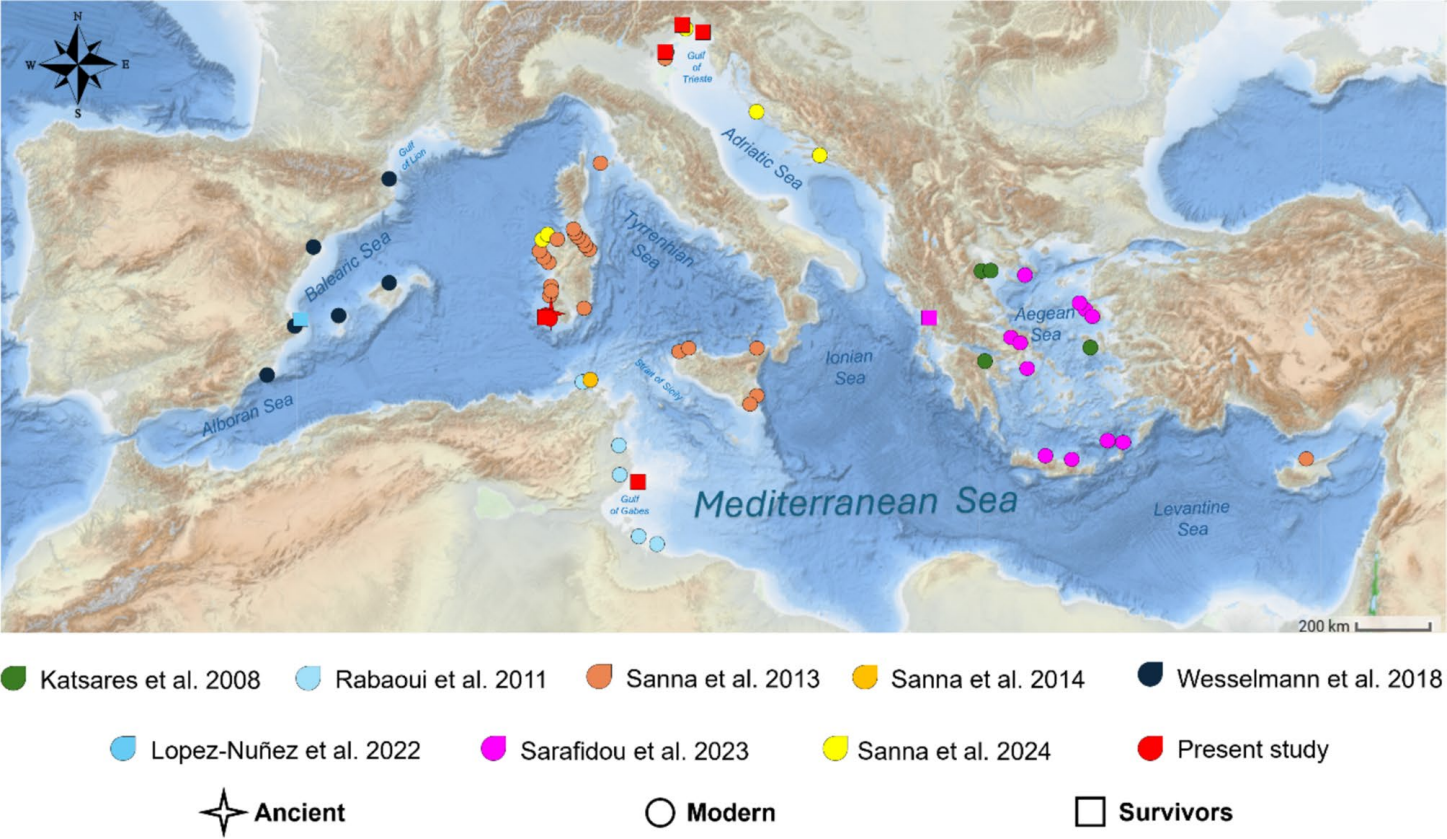
Map of the sampling sites. The map shows the geographical locations for the sequences obtained in the present study along with those from previous research^6,7,10,11,14,24,32,33^. The different studies are indicated using distinct colours, while unique symbols represent key time periods: ancient populations (300 years ago, 1920s, 1970s, 1990s), modern populations (early 2000s, pre-MMEs), and survivor populations (post-MMEs). Detailed symbol and colour designations are provided in the map legend. The map was generated using the online website ScribbleMaps, freely available at the site https://www.scribblemaps.com/. Minor edits, such as adding labels, symbols and North arrow, were performed using Microsoft PowerPoint (Office 16). Credits: Ilenia Azzena.

The dataset analysed in this study comprises all sequences of *Pinna nobilis* collected before, during, and after the MMEs, either sourced from GenBank^6,7,10,11,14,24,32,33^ (n = 548, last updated on 8th December 2024) or generated in this study (n = 119) from both fresh tissues and museum specimens (1700s -2024) (see sampling details in Fig. 4 and Supplementary Table S1).

For living individuals from Western Mediterranean and North Adriatic Sea, a non-lethal sampling was conducted following the methodology of Sanna et al.^11^. This method was officially approved by the Italian "Istituto Superiore per la Protezione e la Ricerca Ambientale (ISPRA)" and the "Ministero dell’Ambiente e della Tutela del Territorio e del Mare", as reported in the Ethics & Inclusion Statement.

A non-invasive sampling method^24^ was also employed, using a cotton swab to scrape tissue and mucus from the interior surfaces of the species. However, this technique yielded ~ 10 times less DNA than tissue sampling.

### Ancient byssus samples and standardization of extraction protocol

We extracted total genomic DNA from biological tissue fragments belonging to the byssus gland that were still adherent to the basal portion of the keratinous filaments. These old byssus samples sourced from various collections, including samples from Sant’Antioco. These latter included samples donated by the sea silk master Chiara Vigo, the Pes sisters (via Italo Diana’s family), as well as samples from Efisia Murroni’s collection, held in the Museo Etnografico of Sant’Antioco. Additionally, we included byssus samples donated by local fishermen to the same museum.

Since previous inclusive phylogeographic studies on *Pinna nobilis* highlighted the species’ effective dispersal capability across the Mediterranean^11^ and the genetic homogeneity between the Western and Adriatic basins^14^, ancient samples from Sant’Antioco Island can be considered as part of a representative population of Western Mediterranean. In this way, we assume to have access not only to ancient *Pinna nobilis* individuals from Sant’Antioco Island, but to a substantial representation of entire Western Mediterranean populations from key time periods: 1920s, 1970s, and 1990s.

A standardized protocol was developed using the Macherey–Nagel Nucleo Spin Tissue Kit (MACHEREY–NAGEL GmbH & Co. KG; Neumann Neander Str. 6–8 D-52355 Düren, Germany). Since the lack of prior studies on DNA extraction from byssus, we conducted tests using two (SAV1 and SAV2) samples (see Table S2 for the full list of hydrating and incubation time conditions) to optimize the procedure.

These tests were conducted using various extraction combinations on both dry and rehydrated tissues samples (~ 25 mg), with rehydration performed using Milli-Q water. Additionally, we tested different incubation times for the action of the proteolytic enzyme, proteinase K (see Table S2 for the full list of hydrating and incubation time conditions).

These trials were performed with the aim of identifying the most efficient method for extracting DNA from old biological tissue fragments belonging to the byssus gland still adherent to the basal portion of filaments. After completing the initial phase of experimentation, we moved forward with the protocol as recommended by the manufacturers, making only a single variation: rather than eluting the final extract in 100 μl of elution buffer, we opted for 50 μl to obtain a more concentrated DNA solution.

The DNA concentration obtained for the samples were quantified using the Nanodrop™ Lite Spectrophotometer (Thermo Scientific, Waltham, MA, USA) (see Table S3 for the protocol details and DNA yield).

All the combinations were tested through standard PCR with species-specific primers^11^ for Cytochrome c Oxidase subunit I of *Pinna nobilis*. The test (number 5 in Table S2), which contemplate 5 h of hydration + 2 h of incubation at 56 °C, produced the most reliable results, yielding average concentrations of 2.5 ng/µl (see Table S3).

Although some other combinations provided higher DNA concentrations, they simultaneously exhibited lower absorbance values (see Table S3 for details), and the electrophoresis did not display bands or, if yes, the bands did not correspond to the expected base pair (bp) size (see Figure S2).

It is worth noting that for the dry or highly hydrated attempts (e.g., tests 1, 2, 3, 6, and 7 in Table S2), the PCR products which were twice the expected size (as shown in Figure S2). Subsequent sequencing reactions and BLAST analyses confirmed the presence of fungal amplification, specifically belonging to the *Aspergillus* genus. These findings further emphasize the effectiveness of test number 5 (5 h of hydration + 2 h of incubation at 56 °C, see Table S2), in obtaining reliable DNA extracts from old biological samples of byssus gland.

### Molecular analyses

For living individuals total genomic DNA was extracted from both mantle tissue (sampling method from Sanna et al.^11^) and cotton swab (sampling method from Sarafidou et al.^24^) using the Macherey–Nagel NucleoSpin Tissue Kit (MACHEREY–NAGEL GmbH & Co. KG, Düren, Germany) following the manufacturer’s instructions. DNA was quantified with a Nanodrop™ Lite Spectrophotometer (Thermo Scientific, Waltham, MA, USA), yielding average concentrations of: (i) 79 ng/µl for the tissue sampling method and (ii) 7.5 ng/µl for the cotton swab sampling method. The mitochondrial Cytochrome c Oxidase subunit I gene (COI) was partially amplified using standard PCR with species-specific primers (L: 5′-GGTTGAACTATHTATCCNCC-3′ and H: 5′-GAAATCATYCCAAAAGC-3′) following the protocol used in Sanna et al.^11^, producing a 338 base pair fragment. Electrophoresis was performed on 2% agarose gels in 1 × TAE buffer (Tris–acetate-EDTA, pH 8.3) stained with GelRed Nucleic Acid Stain (Biotium Inc., Fremont, CA, USA). PCR products were purified using ExoSAP-IT (USB Corporation, Cleveland, OH, USA) and sequenced in both forward and reverse directions using the same PCR primers through an external Sanger sequencing service (Macrogen Europe, Amsterdam, The Netherlands, and Macrogen Europe, Milano, Italy).

### Phylogenetics and phylogeographic analyses

The 119 newly generated sequences (GenBank PQ728133-PQ728251, see Table S1 for details) were aligned with all previously deposited sequences in GenBank^6,7,10,11,14,24,32,33^ utilizing Clustal Omega^38^.

Genetic variation within datasets was assessed using DnaSP version 6.12.03^39^.

The best probabilistic model of sequence evolution was determined using jModeltest 2.1.3^40^, identifying GTR + G^41^ as the best-fit model.

Phylogeographic and phylogenetic analyses included a comprehensive dataset spanning all *Pinna nobilis* sequences from the 1700s to 2024 (see Table S1) to evaluate potential diachronic variations in genetic variability in response to human and environmental stressors.

Genetic relationships between haplotypes and their distribution frequencies were assessed through a median-joining network^42^ using Network 10.2.0.0 (www.fluxus-engineering.com).

To assess the presence of isolation by distance (IBD), a Mantel test was performed using Pearson’s correlation to evaluate the relationship between genetic and geographic distances. Genetic distances were computed from the DNA sequence alignment using the APE package v.5.8-1^43^ in R v.4.3.1^44^, applying the “raw” model (p-distance). Geographic distances were calculated as Euclidean distances (in meters) between sampling site coordinates^45^ using the GEOSPHERE package v.1.5-20^46^. The Mantel test, performed with the VEGAN package v.2.6-10^47^, using 10,000 permutations to assess statistical significance. The strength and significance of the association were evaluated using the Mantel statistic (r) and its associated p-value. Spatial Principal Component Analysis (sPCA) was performed by using ADEGENET package v.2.1.22^48^ in order to detect spatial patterns of genetic variation, including both global structures (e.g., gradients) and local structures (e.g., genetic discontinuities), by incorporating spatial information into multivariate analysis. Positive values of Moran’s I (Moran 1950) ^49^ indicate positive spatial autocorrelation, where nearby individuals tend to share similar genetic profiles. In contrast, negative values reflect negative spatial autocorrelation, suggesting sharp local genetic discontinuities between geographically close individuals, potentially due to barriers to gene flow or local isolation. Values near zero indicate no spatial structure, corresponding to a random spatial distribution of genetic variation.

### Molecular dating and estimation of the divergence time analyses

An additional dataset was created by selecting the 100 haplotypes identified within the whole dataset of 667 sequences by means of DnaSP 6.12.03^39^. Subsequently, this subset was used to estimate the divergence time of each lineage found.

Phylogenetic relationships among haplotypes were inferred through Bayesian Inference (BI) using MrBayes 3.2.6^49^ following Scarpa et al.^50^.

Divergence times of the clades, identified in the Cytochrome c Oxidase subunit I haplotypes phylogenetic tree, were estimated with BEAST 1.10.4^51^ applying evolutionary rates for marine bivalves with pelagic larval dispersal as suggested by Luttikhuizen et al.^52^ and recently verified by Sanna et al.^14^. In BEAUti (part of the BEAST package), mutation rates were specified using a normal distribution ranging from 0.14 to 0.52% divergence per site per million years. Substitution models were defined based on the results of jModeltest, setting the model to GTR with estimated base frequencies, a Gamma distribution plus invariant sites for heterogeneity, and four gamma rate categories. A lognormal uncorrelated relaxed clock model was chosen to allow for rate variation among branches, assuming evolutionary rates vary independently across the tree. The Speciation Yule Process^53,54^ was used as the tree prior to model a constant speciation rate. Model priors and parameters were tailored to calibrate the time-tree using the mutation rates per million years, with divergence estimates derived from a normal distribution reflecting minimum, mean, and maximum mutation rate values. Operator settings followed the BEAST user manual guidelines. The lognormal uncorrelated relaxed clock model also provided insight into how clock-like the data were, using the ucld.stdev parameter: values close to zero indicated a clock-like pattern, while values well above one suggested considerable rate heterogeneity across lineages. To ensure sufficient sampling of all model parameters (ESS > 200), the analysis was run for 400 million generations, with tree samples taken every 40,000 generations, following the protocol of Scarpa et al.^55^. Tracer v1.7.1^56^ was employed to inspect the log files, verify convergence, check ESS values, and estimate node ages. Final visualization and editing of the time-calibrated phylogenetic tree were carried out using TreeAnnotator v1.10.4 and FigTree v1.4.4 (http://tree.bio.ed.ac.uk/software/figtree/).

## Supplementary Information

Supplementary Information.

## Data Availability

The datasets used and analysed during the current study are available from the corresponding author (Daria Sanna darsanna@uniss.it) on reasonable request. The new sequences generated and analysed during the current study are available in the GenBank repository under the accession numbers PQ728133-PQ728251.These sequences were deposited on December 10, 2024 (SUB14920067) and are set for release on March 18, 2026, or upon manuscript publication. GenBank accession numbers for all data analysed during this study, except for newly generated sequences, are included in this published article [and its supplementary information files, Table S1].
