# Supplementary material for: Conservation hints for Pinna nobilis from a century-old genetic time capsule
Source: Sci Rep. 2025 Oct 28;15:37660. doi: 10.1038/s41598-025-21574-6 (PMC12569023; doi:10.1038/s41598-025-21574-6)

**Supplementary Materials.**

**Table S1.** The table provides data on the sampling collection. Details on the sites for each geographic area where *Pinna nobilis* individuals were collected during this study are included for ancient, modern, and survivor populations. It also provides details of GenBank sequences of *Pinna nobilis* from various locations across the Mediterranean, which were used in the analyses. Accession numbers marked with an asterisk (*) indicate instances where only the haplotypes are accessible in GenBank.

| **ANCIENT** | | | | | |  |
| --- | --- | --- | --- | --- | --- | --- |
| **Sardinia** | | | | | |  |
| **Sample Code** | **Sampling Year** | **Sampling Area** | **# Specimens** | **GenBank Code** | **Paper** |  |
| SAV48 | ≈ 1700s | Sant’Antioco | 1 | PQ728176 | Present study |  |
| SAV1-18 | 1920’s |  | 17 | PQ728133 - PQ728149 |  |  |
| SAV19-39 | 1970’s |  | 20 | PQ728150 - PQ728169 |  |  |
| SAV40-46 | 1990’s |  | 6 | PQ728170 - PQ728175 |  |  |
| **MODERN** | | | | | |  |
| **Sardinia** | | | | | |  |
| **Sample Code** | **Sampling Year** | **Sampling Area** | **# Specimens** | **GenBank Code** | **Paper** |  |
| OSR | 2013 | Ossario (Asinara) | 12 | OR782596 - OR782633 | Sanna et al. 2024 |  |
| ASI | 2015 | Cala di Scombro di Dentro and Cala Reale (Asinara) | 38 | OR782634 - OR782645 |  |  |
| SAN | 2019 | Sant’Antioco | 11 | PQ728177-PQ728180  PQ728192 - PQ728198 | Present study |  |
| CAL | 2019 | Calasetta (Sant’Antioco) | 11 | PQ728181 - PQ728191 |  |  |
| BPC | 2010 | Baia di Porto Conte | 18 | JX854788 - JX854805 | Sanna et al. 2013 |  |
| POR |  | Torre del Porticciolo | 3 | JX854806 - JX854808 |  |  |
| LAZ |  | Lazzareto | 2 | JX854809 - JX854810 |  |  |
| OSM |  | Ospedale Marino | 21 | JX854811 - JX854831 |  |  |
| MOL |  | Molara | 11 | JX854832 - JX854842 |  |  |
| CCE |  | Capo Ceraso | 13 | JX854843 - JX854855 |  |  |
| MPE |  | Monte Petrosu (Sassi piatti and Isola Cava) | 4 | JX854861 - JX854864 |  |  |
| SAL | 2011 | Le Saline | 5 | JX854856 - JX854860 |  |  |
| OTT |  | Porto Ottiolu | 5 | JX854865 - JX854869 |  |  |
| ORI |  | Oristano | 10 | JX854870 - JX854879 |  |  |
| MAR |  | Marceddì | 5 | JX854880 - JX854884 |  |  |
| IMV |  | Isola di Mal di Ventre | 4 | JX854885 - JX854888 |  |  |
| VMS |  | Villasimius (Capo Caterina) | 4 | JX854889 - JX854892 |  |  |
| CPA |  | Costa Paradiso | 5 | JX854893 - JX854897 |  |  |
| MAD |  | La Maddalena Island (Cala Camiciotto) | 18 | JX854898 - JX854915 |  |  |
| **Corsica** | | | | | |  |
| IPI | 2011 | Isola Piana | 13 | JX854916 - JX854928 | Sanna et al. 2013 |  |
| CPC |  | Cala Pesciu Cane | 12 | JX854929 - JX854940 |  |  |
| **Elba Island** | | | | | |  |
| ELB | 2011 | Capo Enfola | 10 | JX854992 - JX855001 | Sanna et al. 2013 |  |
| **Sicily** | | | | | |  |
| SVC | 2011 | San Vito lo Capo (Secca di Cala Rossa) | 7 | JX854941 - JX854947 | Sanna et al. 2013 |  |
| MON |  | Mondello | 11 | JX854948 - JX854958 |  |  |
| MLZ |  | Milazzo | 10 | JX854959 - JX854968 |  |  |
| PAC |  | Pachino (Capo Passero) | 8 | JX854969 - JX854976 |  |  |
| OGN |  | Ognina di Siracusa | 15 | JX854977 - JX854991 |  |  |
| **Adriatic** **Sea** | | | | | |  |
| VEN | 2011 | Ottagono Alberoni and Santa Maria del Lago | 20 | JX855002 - JX855021 | Sanna et al. 2013 |  |
| MIR | 2018 | Miramare (Gulf of Trieste) | 18 | OR782678 - OR782695 | Sanna et al. 2024 |  |
| TEL | 2015 | Telašćica - Island Buč | 14 | OR782646 - OR782659 |  |  |
| MLJ | 2015 | Mljet - Lake Malo Jezero | 18 | OR782660 - OR782677 |  |  |
| **Cyprus** | | | | | |  |
| CYP | 2011 | Karaoglanoglu | 2 | JX855022 - JX855023 | Sanna et al. 2013 |  |
| **Aegean** **Sea** | | | | | |  |
| EPA - EPT | - | Epanomi | 9 | DQ448216 - DQ448217 | Katsares et al. 2008 |  |
|  |  |  |  | EF536827 - EF536832 |  |  |
| AGG | - | Aggelochori | 9 | EF536833 - EF536841 |  |  |
| XIO | - | Xios Island | 5 | EF536842 - EF536846 |  |  |
| KOR | - | Korinthiakos Gulf | 3 | EF536847 - EF536849 |  |  |
| LAV | 2018 | Lavrio | 2 | OX407247 - OX407248 | Sarafidou et al. 2023 |  |
| IT |  | Tristomo | 4 | OX407243 - OX407246 |  |  |
| ID |  | Diafani | 4 | OX407239 - OX407242 |  |  |
| GT |  | Tristomo | 3 | OX407236 - OX407238 |  |  |
| GD |  | Diafani | 6 | OX407230 - OX407235 |  |  |
| D |  | Diafani | 3 | OX407227 - OX407229 |  |  |
| TS 4-6 |  | Kalloni | 3 | OX407224 - OX407226 |  |  |
| TS 1-3 |  | Gera | 3 | OX407221 - OX407223 |  |  |
| X |  | Tristomo | 8 | OX407188 - OX407195 |  |  |
| A |  | Astakida | 5 | OX407183 - OX407187 |  |  |
| MYT | 2019 | Kalloni | 9 | OX407212 - OX407220 |  |  |
| VOUR |  | Vourvourou | 2 | OX407181 - OX407182 |  |  |
| OR |  | Oropos | 4 | OX407177 - OX407180 |  |  |
| AV |  | Avlida | 2 | OX407175 - OX407176 |  |  |
| BAL |  | Bali | 1 | OX407174 |  |  |
| EL |  | Elounda | 2 | OX407172 - OX407173 |  |  |
| **Tunisian coastlines** | | | | | |  |
| N | - | Bizerta Lagoon | 7 | HM998857  -  HM998866* | Rabaoui et al. 2011 |  |
| M | - | Monastir (Stah Jaber) | 9 |  |  |  |
| S | - | Kerkennah Island | 7 |  |  |  |
| B | - | El Bibane Lagoon | 9 |  |  |  |
| K | - | El Ketef | 17 |  |  |  |
| BIZ | 2013 | Bizerta Lagoon | 1 | KF612603 | Sanna et al. 2014 |  |
| **French** **coastlines** | | | | | |  |
| BAN | 2014 | Banyuls | 9 | KY321755 - KY321811 | Wesselmann et al. 2018 |  |
| **Spanish coastlines** | | | | | |  |
| IBI | 2011 | Ibiza | 10 | KY321755 - KY321811 | Wesselmann et al. 2018 |  |
| MALL |  | Mallorca | 9 |  |  |  |
| EBR | 2014 | Ebro Delta | 9 |  |  |  |
| MUR |  | Murcia | 10 |  |  |  |
| ALI |  | Alicante | 10 |  |  |  |
| **SURVIVOR** | | | | | | |
| **Spain** | | | | | | |
| **Sample Code** | | **Sampling Year** | **Sampling Area** | **# Specimens** | **GenBank Code** | **Paper** |
| PN | | 2020 | Mar Menor | 2 | OM397480 - OM397481 | Lopez-Nuñez et al. 2022 |
| **Sardinia** | | | | | | |
| SAN | | 2024 | Sant’Antioco | 10 | PQ728242 - PQ728251 | Present study |
| **Tunisia** | | | | | | |
| KER | | 2022 | Kerkennah Islands | 3 | PQ728204 - PQ728206 | Present study |
| **North Adriatic Sea** | | | | | | |
| MIR | | 2022 - 2023 | Miramare (Trieste, Italy) | 10 | PQ728199 - PQ728203  PQ728237 - PQ728241 | Present study |
| TRI | |  | Trieste (Italy) | 15 | PQ728207 - PQ728221 |  |
| CAM | |  | Venetian Lagoon (Italy) | 12 | PQ728222 - PQ728233 |  |
| SLO | |  | Slovenia | 3 | PQ728234 - PQ728236 |  |
| **Greece** | | | | | | |
| AMV | | 2021 | Amvrakikos Gulf | 16 | OX407196 - OX407211 | Sarafidou et al. 2023 |

**Table S2.** Table of hydration and incubation time conditions tested for optimizing DNA extraction from byssus. The hyphen "–" symbol indicates samples that remained dry without hydration.

| **Test** | **Hydration time** | **Incubation time at 56°C** |
| --- | --- | --- |
| 1 | - | 1 h |
| 2 | - | 3 h |
| 3 | - | overnight |
| 4 | 3 h | 3 h |
| 5 | 5 h | 2 h |
| 6 | 7 h | overnight |
| 7 | 72 h | 2 h |
| 8 | 30 min (4 cycles) | 2 h |

**Table S3.** Quantification results for DNA extractions from byssus. The table shows the values obtained for each combination of hydration and incubation conditions. The hyphen " - " represents failed attempts.

| **Sample ID** | **Hydration time** | **Incubation time** | **DNA (ng/μl)** | **A 260/280** |
| --- | --- | --- | --- | --- |
| SAV1 | 0 h. | 1 h. | 6.8 | 1.45 |
| SAV2 |  |  | 2.8 | 1.36 |
| SAV1 | 0 h. | 3 h. | 8.6 | 1.59 |
| SAV2 |  |  | 6.1 | 1.46 |
| SAV1 | 0 h. | overnight | - | - |
| SAV2 |  |  | 15.7 | 1.26 |
| SAV1 | 3 h. | 3 h. | 2.8 | 1.68 |
| SAV2 |  |  | 1.6 | 1.40 |
| SAV1 | 5 h. | 2 h. | 2.8 | 1.65 |
| SAV2 |  |  | 2.3 | 1.58 |
| SAV1 | 7 h. | overnight | 5.1 | 1.32 |
| SAV2 |  |  | 3.5 | 1.28 |
| SAV1 | 72 h. | 2 h. | 8.0 | 1.45 |
| SAV2 |  |  | 5.8 | 1.33 |
| SAV1 | 4 x 30 min. | 2 h. | 7.4 | 1.39 |
| SAV2 |  |  | 6.2 | 1.33 |


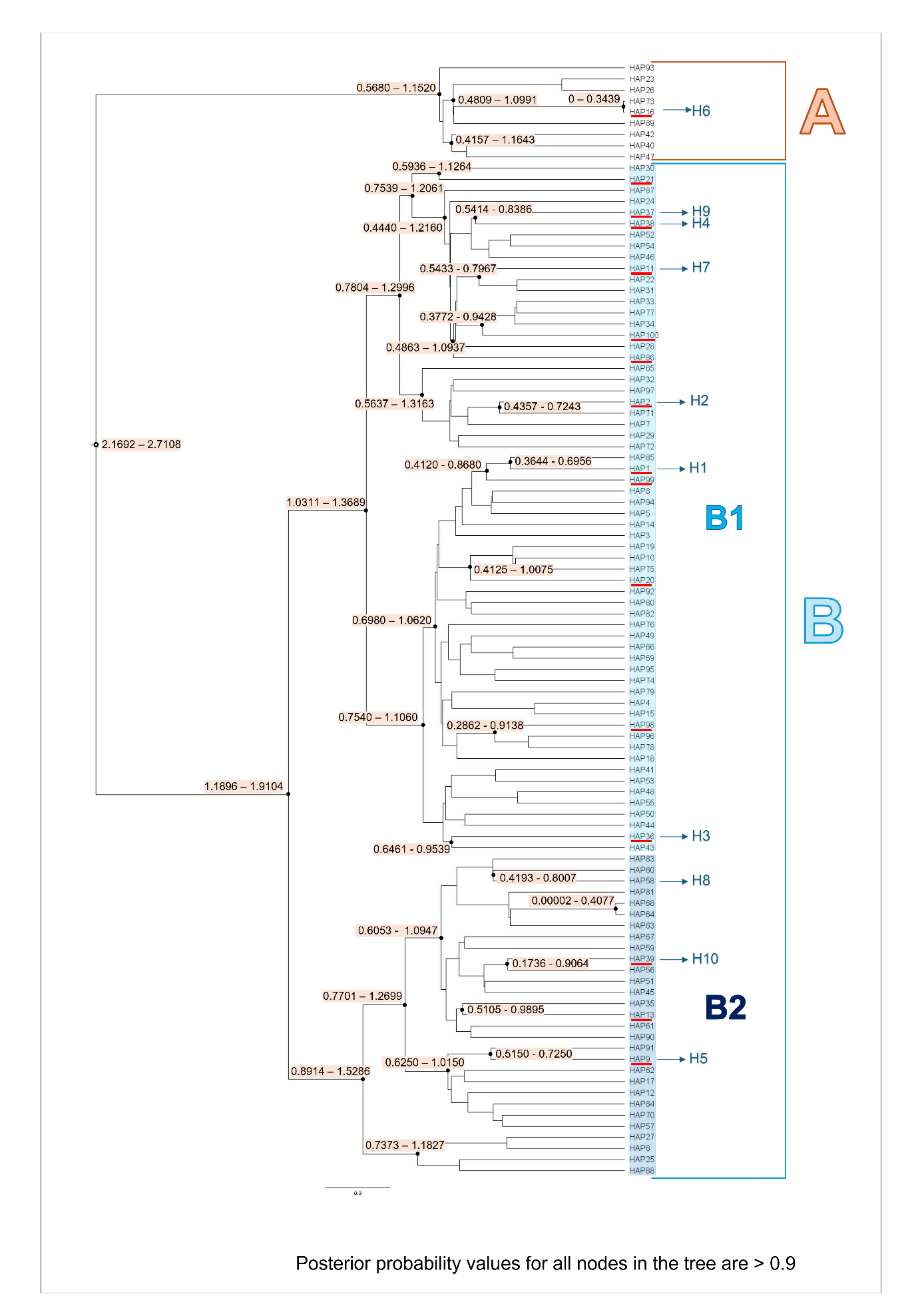
**Figure S1.** Time-calibrated phylogenetic tree of *Pinna nobilis* COI haplotypes based on a subset of 100 sequences. Branch lengths represent estimated divergence times, and 95% Highest Posterior Density (HPD) intervals are indicated by at each node.

**Figure S2.** The image shows the results of electrophoresis testing at different hydration states for the same individual, SAV1. Wells 1 and 2 (representing Test 1 and Test 7, respectively) display a fragment approximately twice the expected size, while well 6 (Test 5), used as positive control, shows the expected band around 400 base pairs (bp).


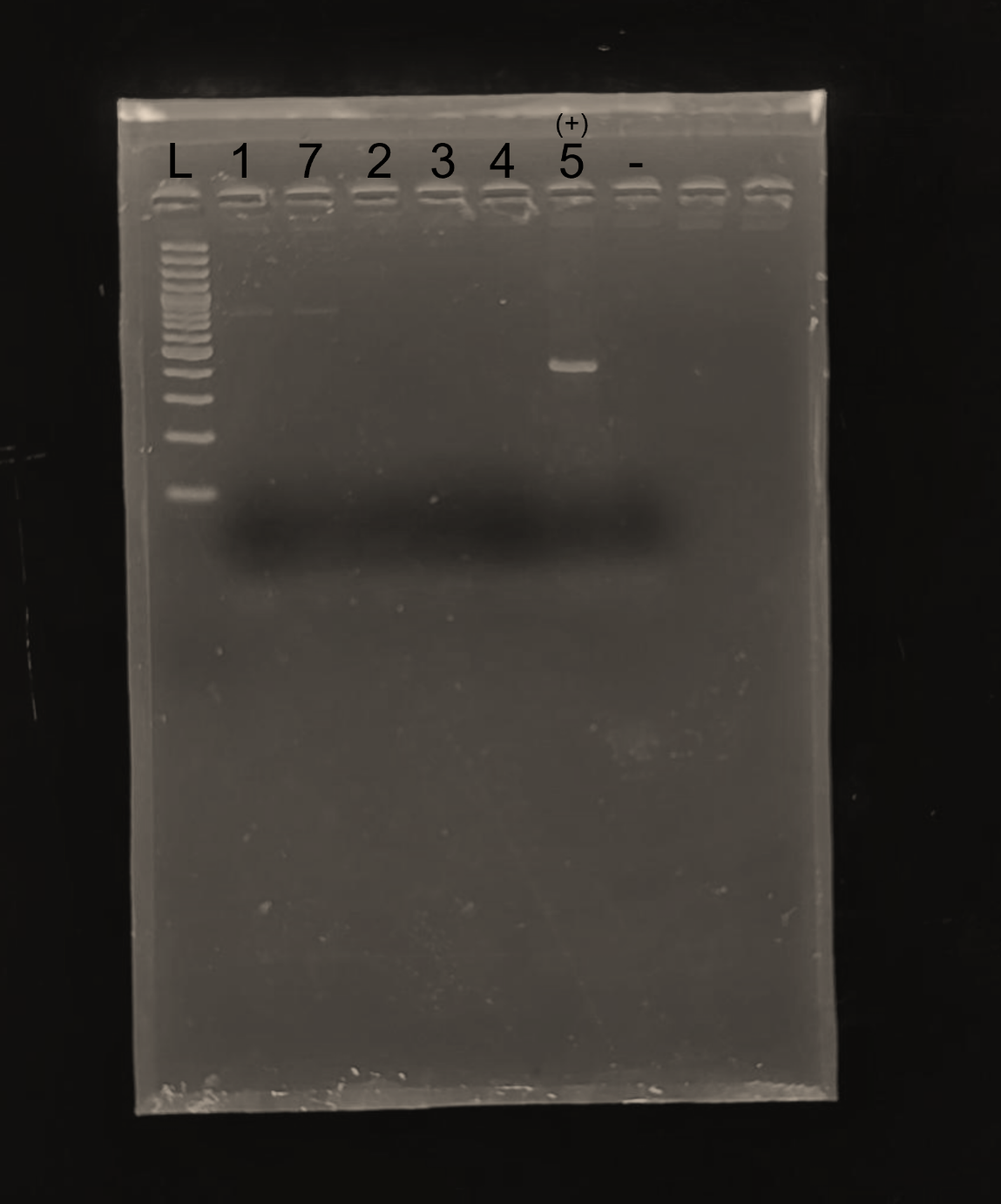

Supplement: Supplementary file 1 — Supplementary Information. [file 41598_2025_21574_MOESM1_ESM.docx]
